# Grouping crossbred Holstein x Gyr heifers according to different feed efficiency indexes and its effects on energy and nitrogen partitioning, blood metabolic variables and gas exchanges

**DOI:** 10.1371/journal.pone.0238419

**Published:** 2020-09-11

**Authors:** Danieli Cabral da Silva, Luiz Gustavo Ribeiro Pereira, Juliana Aparecida Mello Lima, Fernanda Samarini Machado, Alexandre Lima Ferreira, Thierry Ribeiro Tomich, Sandra Gesteira Coelho, Rogério Martins Maurício, Mariana Magalhães Campos

**Affiliations:** 1 Department of Animal Science, State University of Southwestern Bahia (UESB), Bahia, Brazil; 2 Brazilian Agricultural Research Corporation–Embrapa Dairy Cattle, Juiz de Fora, Minas Gerais, Brazil; 3 Department of Animal Science, School of Veterinary Medicine, Federal University of Minas Gerais (UFMG), Minas Gerais, Brazil; 4 Federal University of São João del-Rei, Minas Gerais, Brazil; Universidade Federal de Viçosa, BRAZIL

## Abstract

The objectives of this study were: *i*) to classify animals into groups of high and low feed efficiency (FE) using three FE indexes (Residual feed intake (RFI), Residual weight gain (RG) and Feed conversion efficiency (FCE)), and *ii*) to evaluate whether crossbreed Holstein x Gyr heifers divergent for FE indexes exhibit differences in nutrient intake and digestibility, energy partitioning, heat production, methane emissions, nitrogen partitioning and blood parameters. Thirty-five heifers were housed in a tie-stall, received *ad libitum* TMR (75:25, corn silage: concentrate) and were ranked and classified into high (HE) or low efficiency (LE) for RFI, RG and FCE. The number of animals for each HE group were 13 (< 0.5 standard deviation (SD) for RFI, 11 for RG and 11 for FCE (> 0.5 SD) and for the LE were 10 (> 0.5 SD) for RFI, 11 for RG and 12 for FCE (< 0.5 SD). Gas exchanges (O_2_ consumption, CO_2_ and CH_4_ production) in open-circuit respiratory chambers and whole tract digestibility trial was performed. A completely randomized experimental design was used and the data were analyzed by ANOVA and correlation study. High efficiency animals for RFI produced less CO_2,_ consumed less O_2_ and had lower heat production (HP). Methane production was positively correlated with RFI. High efficiency RG had higher O_2_ consumption and CO_2_ production in relation to LE-RG. High efficiency FCE had greater NFC digestibility, higher positive energy balance (EB) and excreted (11.4 g/d) less nitrogen in urine. High efficiency RG and FCE groups emitted less CH_4_ per kg of weight gain than LE animals. Animals HE for RFI and FCE had lower β-hydroxybutyrate and higher glucose concentrations, respectively. The differences in intake, digestibility, energy and nitrogen partition, CH_4_ emission, blood metabolic variables and heat production between the HE and LE groups varied according to the efficiency indexes adopted. The HP (kcal/d/BW^0.75^) was lower for HE animals for RFI and FCE indexes.

## Introduction

Increasing the efficiency of livestock production systems is essential to improve productivity and to reduce negative environmental impacts. Feed efficiency (FE) is a highly important economic trait in milk production systems[1,2]. For growing cattle, the most efficient animals have lower production costs[3,4]. According to Arthur and Herd[4], there is individual variation in nutrient utilization efficiency among animals with similar characteristics. Differences in efficiency between animals can be dependent on body weight, stage of production, growth composition, environmental conditions and their interactions with other factors such as rumen kinetics, digestion, absorption and efficiency of energy and protein utilization[5,6]. However, the relationships between groups of high and low FE and the effects on energy and nitrogen partitioning, blood metabolic variables and gas exchanges are not well understood.

Feed conversion efficiency (FCE) is a traditional efficiency index in dairy cattle[7,8]. On the other hand, the residual feed intake (RFI), predominantly used in beef cattle [9,10], has also provided relevant data about the efficiency of *Bos taurus taurus* dairy cows[11,12]. The RFI is calculated by the difference between actual and expected feed intake of a group of animals over a defined period of time [13]. Residual weight gain (RG) is not a usually index for dairy cattle, but may be interesting due to allow a selection of animals with better average daily gain without increasing dry matter intake [14]. There is a correlation between body weight and age at puberty [15], thus, the use of RG can affect indirectly the age of puberty, which tends to be later in Zebu crossbred animals.

Long-term and high-cost experimental trials are required to obtain FE indexes [16]. Therefore, the search for traits that works as biomarkers of FE (e.g. blood metabolic variables, infrared thermography and feeding behavior) has grown in recent years and may allow for the development of low cost ways to identify efficient animals [17,18].

Thus, the objectives of this study were: *i*) to classify animals into groups of high and low FE using three FE indexes (RFI, RG and FCE), and *ii*) to evaluate whether crossbreed Holstein x Gyr heifers divergent for FE indexes exhibit differences in nutrient intake and digestibility, energy partitioning, heat production, methane emissions, nitrogen partitioning and blood parameters.

So, the hypothesis of the present study were: i) Despite of which FE index used to classify animals in HE and LE, the groups will present physiological differences that justify a better use of energy and protein in HE group, and ii) Classifications by different feed efficiency indexes will result in different physiological responses between animals classified HE and LE.

## Material and methods

This study was performed at the Multi-use Complex on Livestock Bioefficiency and Sustainability of Brazilian Agricultural Research Corporation (Embrapa), located in Coronel Pacheco, Minas Gerais, Brazil. All procedures used were approved by the Ethics Committee of Embrapa Dairy Cattle (number: 05/2015).

### Feed efficiency indexes

Three different FE indexes (RFI, RG and FCE) were measured on a group of thirty-six F1 Holstein x Gyr heifers, averaging 146 ± 28 d (mean ± SD) of age and 152 ± 21.7 kg of initial body weight (BW), in a 113 d pre-experimental study. Heifers were weighed once a week before morning feeding using an electronic scale (Toledo MGR-2000, São Bernardo, Brazil). Average daily weight gain (ADG) was calculated as the linear regression coefficient of BW using PROC REG program from Statistical Analysis Software (SAS Inst. Inc., Cary, NC), composed of 14 BW measurements per heifer at 7 d intervals, and metabolic body weight (BW^0.75^) was calculated using the BW on day 56 of the pre-experimental study.

Dry matter intake, BW^0.75^, and ADG were used to estimate RFI and RG using linear regressions[19], where RFI and RG were calculated as the differences between actual and predicted dry matter intake (DMI) and ADG, respectively, as follows:

$$Yj=\beta 0+\beta 1\left(BW^{0.75}j\right)+\beta 2\left(ADGj\;or\;DMIj\right)+ej,$$

where Yj is the standardized DMI (RFI) or ADG (RG) of animal j, β0 is the intercept, β1 is the regression coefficient for BW^0.75^, β2 is the regression coefficient for ADG (RFI) or DMI (RG), and ej is the error term for animal j.

Feed conversion efficiency was measured using the relationship between mean ADG and daily DMI.

Thirty-five heifers were classified into two RFI, RG and FCE groups: high efficiency (HE) and low efficiency (LE). The animals were ranked based on a previous FE assay (before the digestibility and metabolism trial) with 36 animals, but one of the animals was excluded due to leg fracture. The excluded animal had been classified as intermediary for RFI, RG and FCE and it was not used in the digestibility and metabolism study. The original groups were made based on SD. The number of animals per treatment for the HE group were 13 (< 0.5 SD) for RFI, 11 for RG and 11 for FCE (> 0.5 SD). For the LE group the number of animals were 10 (> 0.5 SD) for RFI, 11 for RG and 12 for FCE (< 0.5 SD). The other 12, 13 and 12 animals were classified as intermediary for RFI, RG and FCE, respectively and were not included in subsequent analyses. As the distribution of the animals followed a normal distribution, we chose to balance the groups to roughly equal size of n = 12 for HE and LE groups. The percentage of animals that had the same classification for FE as HE or LE indexes were 57% comparing RG and FCE, 37% comparing RFI and FCE and 14% comparing RFI and RG. Pre-experimental FE indexes (RFI, RG and FCE), DMI, BW and ADG of the high and low efficiency groups are presented in Table 1. The data of individual classification for the different FE groups are presented in supplementary material.

**Table 1:** Means of pre-experimental feed efficiencies^1^ index (RFI, RG and FCE) for the dairy heifers classifieds as high (HE) or low efficient (LE).

Traits (unit)	RFI^2^		SEM^7^	*P*-value^8^	RG^3^		SEM	*P*-value	FCE^4^		SEM	*P*-value
	HE^5^	LE^6^			HE	LE			HE	LE		
RFI (kg/d)	-0.27	0.30	0.07	<0.01	-	-	-	-	-	-	-	-
RG (kg/d)	-	-	-	-	0.09	-0.09	0.02	<0.01	-	-	-	-
FCE (kg ADG^2^/kg DMI)	-	-	-	-	-	-	-	-	0.21	0.15	0.01	<0.01
DMI (kg DM/d)^9^	4.61	5.14	0.15	<0.01	4.80	4.64	0.13	0.21	4.75	4.89	0.14	0.49
BW in the middle of the trial (kg)^10^	202	200	4.36	0.72	200	194	4.84	<0.01	203	192	5.16	<0.01
ADG (kg/d)^11^	0.86	0.87	0.02	0.58	0.95	0.75	0.02	<0.01	0.89	0.82	0.02	<0.01

^1^Selection of the divergent animals for RFI, RG, FCE, was based on data of the feed efficiency test of an earlier study.

^2^Residual feed intake

^3^Residual weight gain

^4^Feed conversion efficiency

^5^High efficiency

^6^Low efficiency

^7^Stander error of the means

^8^Statistical difference between HE and LE

^9^Dry matter intake

^10^Body weigh

^11^Average daily gain

### Heifers, housing and management

Holstein x Gyr heifers (F1) averaged 258 ± 20 days of age and 293 ± 21.5 kg of initial BW at the beginning of the metabolism study. Heifers were housed in individual tie stalls (2.5 x 1.2 m) with bedding by rubber mats (WingFlex, Kraiburg TPE GmbH & Co., Waldkraiburg, Germany). The same total mixed ration (TMR) was offered throughout the entire study, starting with the selection period. Diet DM and crude protein (CP) contents were 43.8% and 175 g/kg DM, respectively, and included (DM basis) 75% corn silage and 25% concentrate (96% soybean meal and 4% mineral premix, DM basis) (Table 2). The daily offered amount of TMR was adjusted to allow 10% orts on an as-fed basis, based on the intake from the previous day. Water was available *ad libitum*. Adjustments to the diet were made twice weekly due to possible changes in silage DM content. Heifers were fed once per day at 0830 h and orts were removed and weighed daily before feeding.

**Table 2:** Ingredients and chemical composition (DM basis, %) of total mixed ration (TMR, 75% corn silage and 25% concentrate).

Item	TMR^1^
***Ingredients***	
Soybean Meal, g/kg	240
Mineral Mix, g/kg^2^	0.10
Corn Silage, g/kg	750
***Chemical composition***	
Dry matter, g/kg	438
Organic matter, g/kg	934
Crude protein, g/kg	175
Ether extract, g/kg	24.1
Neutral detergent fiber, g/kg	422
Acid detergent fiber, g/kg	238
Non-fibrous carbohydrates, g/kg	313
Gross energy, kcal/kg	4084

^1^Total mixed ration

^2^ Mineral mix (Fosbovi 40, MN, DSM^®^ São Paulo/Brazil) contained: 260 g/kg calcium; 174 g/kg phosphorus; 24 g/kg sulphur; 0.10 g/kg cobalt; 1.25 g/kg copper; 1.79 g/kg iron; 0.09 g/kg iodine; 2 g/kg manganese; 0.01 g/kg selenium; 5.27 g/kg zinc and 1.74 g/kg fluorine.

The same 35 heifers were evaluated in the whole tract digestibility assay and gas exchange study with open circuit respiration chambers. Due to the restriction of only four respiration chambers it was necessary to stagger the digestibility and respirometry evaluations. The heifers were divided randomly into four groups (numbers: 9, 9, 9 and 8) without prior knowledge of the FE ranking. The experimental period consisted of 10 d adaptation and 5 d metabolism trial period. The heifers were acclimated to tie-stall and respiration chambers before the metabolism trial began.

### Whole tract digestibility and nitrogen balance

Total feces were collected from days 10 to 14, and total urine were collected 10 to 11 of each experimental period, to estimate digestibility and nitrogen balance. Plastic containers (50 L) properly capped and identified were used to collect the feces individually. The fecal material produced by each animal was weighed twice daily (10 a.m. and 4 p.m.) and after the homogenization of the contents within each plastic container, approximately 500 g of feces were sampled. Aliquots of silage, concentrate and orts were collected daily over 5 consecutive days, and stored at -20 ^o^C for further processing and analyses. After thawing, feed, orts and feces samples were dried in a forced-ventilation oven (55 ^o^C) for 72 h and ground through a 1 mm screen Wiley mill (A. H. Thomas, Philadelphia, PA). Feces and orts were pooled per animal by daily weight based on DM after drying at 55 ^o^C.

Total urine was collected with indwelling Foley catheters (Rüsch Foley catheter, REF 189230, Teleflex Medical Europe Ltd, Co., Westmeath, Ireland) on the first 2 d of fecal collection. Catheters were attached to hoses to carry urine to individual polyethylene containers, which were kept inside the styrofoam boxes filled by ice. After each 24 h collection day, urine weight was measured, homogenized and a 50 g sample was collected and stored at -20 ^o^C for analysis of nitrogen and gross energy (GE).

Corn silage, concentrate, orts and feces samples were analyzed [20] for DM contents (method 930.15), ash (method 924.05), crude protein (CP: method 984.13), ether extract (EE: method 920.39) and NDF by [21] adapted for use in an ANKOM^220^ Fiber Analyzer (Ankom Technology, Fairport, NY) using F57 filter bags (ANKOM®), with addition of 500 μL/g DM of thermostable amylase without sodium sulphite and corrected for ash and nitrogen [22]. Non-fibrous carbohydrates (NFC) concentration was calculated by NFC = 100 - (NDF% + CP% + EE% + ASH%) according to Mertens [23]. Gross energy was determined using an adiabatic calorimeter (IKA—C5000, IKA® Works, Staufen, Germany). The urine samples were analyzed for GE (calorimetric method) and the quantification of nitrogen content by the Kjeldahl method.

To determine the DM intake (DMI) and nutrients, the following equation was used: DMI = (DM offered–ort DM). The digestibility values (g/kg) were determined as a function of the nutrient disappearance, considering the equation: Nutrient digestibility = ((kg DM ingested × % nutrients)—(kg DM feces × % nutrients)) / (kg DM ingested × % nutrient) × 100. Nitrogen balance was calculated according to the equation: nitrogen retained (g/d) = nitrogen ingested—(fecal nitrogen + urinary nitrogen).

### Respiration exchanges and methane emission

After the whole tract digestibility trial, each of the four groups evaluated was transferred to the four open-circuit respiratory chambers for O_2_ consumption and CO_2_ and CH_4_ production measurements [24]. The chambers are independently climate-controlled (relative humidity setting to 60% ± 2% and temperature to 22 ºC ± 1 ^o^C). Each chamber had a volume of 21.10 m^3^ (3.68 × 2.56 × 2.24 m) and was made from steel with double-glazed windows on either side enabling visual contact between animals. A flow generator and mass flow meter continuously pull air from each chamber and a slight negative pressure inside the chamber is ensured. Air from all chambers and ambient air share a common gas analysis and data acquisition system for monitoring O_2_, CO_2_, and CH_4_ concentrations over the entire measurement period, with the cycle time set to 20 min.

After feeding, two sequences of 22 h respiration measurements were obtained per animal, which was randomly allocated to each chamber. Dry matter intake inside the chamber was measured and compared with the average DMI during the digestibility trial of the respective heifer and if intake dropped by more than 5%, the respiration measurement was repeated. The number of times that a respiration measurement had to be repeated were 4, 5, 6 and 7 for groups 1, 2, 3 and 4 respectively. The same procedure applied for the digestibility trial was used to calculate the DMI inside the chamber. The animals were weighed before and after entering the chamber. Over each 22 h measurement period, gas exchange rates obtained for each cycle time were used to calculate the total gas exchanges as the area under the curve, which was extrapolated to a 24 h period.

### Energy partitioning

Gross energy intake (GEI) and daily fecal and urinary energy outputs were obtained by multiplying DMI, fecal and urinary DM excretion with their respective energy content. Digestible energy intake (DEI) was calculated by the difference between GEI and fecal energy excretion. Metabolizable energy intake (MEI) was derived as the difference between DEI and the sum of urine energy and CH_4_ energy, which was assumed to be 9.45 Kcal/L [25]. Energy retention was calculated as the difference between MEI and heat production. Heat production (HP; Mcal/d) was determined based on measurements of O_2_ consumption (L/d), CO_2_ and CH_4_ production (L/d), and urine nitrogen output (g/d) applying the equation of Brower [25].

### Blood metabolic variables

Blood was sampled after the digestibility trial, 4 h after TMR was offered, by puncture of the coccygeal vein into two 5 mL Vacutainer tubes (Becton, Dickinson and Company, New Jersey, USA) after local antisepsis. To assess circulating insulin and β-hydroxybutyrate (BHB), blood samples were collected into tubes without anticoagulant; and for glucose concentrations, samples were collected into Vacutainer tubes containing sodium fluoride. Tubes were placed in crushed ice until centrifugation at 1.800 × g for 10 min at room temperature (22–25°C). Plasma or serum aliquots were stored at −20°C until analysis. Plasma glucose was measured on an Eon^TM^ microplate spectrophotometer (Biotek Instruments Inc., Winooski, VT, USA) using an enzymatic colorimetric method (Kovalent do Brasil Ltda., Rio de Janeiro, Brazil). The determination of BHB was performed using enzymatic kinetic kit RANBUT—Ref.: RB 1007 (RANDOX Laboratories—Life Sciences Ltd, Crumlin, UK) in an Automatic System for Biochemistry spectrophotometer (Model BIOPLUS BIO 2000®). The intra-assay and inter-assay CV were ≤ 3%. Insulin was analyzed using a bovine ELISA kit (Mercodia, Uppsala, Sweden). The intra-assay and inter-assay CV were ≤ 7.0 and 8.2%, respectively.

### Statistical analysis

Statistical analysis was performed using SAS software (version 9.4, SAS Institute Inc., Cary, NC). Data relative to apparent digestibility, nitrogen balance, energy partitioning and blood parameters were analyzed by ANOVA.

Data relative to DMI, performance, gas exchanges, CH_4_ emission and HP were analyzed as repeated measures using a mixed model procedure. The statistical model included fixed effects of efficiency group, time (week or day) and efficiency group × time interaction as a covariate. The experimental week or day (two sequences of 22 h respiration measurements) was included as a repeated statement and the animal was nested within treatment as a random effect, according to the model:

$$Y_{ijk}=\mu +\tau_{i}+wk+{\left(\tau \times w\right)}_{ik}+\delta_{ij}+\varepsilon_{ijk},$$

where:

Y_*ijk*_ = the dependent variable;

μ = overall mean;

τ_i_ = fixed effect of efficiency group;

wk = fixed effect of repeated measure (day or week);

(τ x w)_ik_ = fixed effect of interaction between group and repeated measure;

δ_ij_ = random error between animals within treatment;

ε_ijk_ = random error between measurements within animals.

The best covariance structure for repeated measures was chosen by the lower corrected Akaike information criteria (AICc).

Means, standard error of the mean, normality and homogeneity of variance were evaluated for all variables using the UNIVARIATE procedure. The comparison between the means of the high (n = 12) and low (n = 12) efficiency groups for the evaluated parameters (productive, nutritional, physiological and metabolic) at different FE indexes were compared by Fisher’s test. Data were considered statistically significant when *P* ≤ 0.05 and a trend towards significance was considered when 0.10 ≥ *P* ˃ 0.05 for all the statistical analyses performed. Pearson’s correlation analyses were performed for each FE index (RFI, RG and FCE). The response variables were conducted by the SAS CORR procedure, considering *P* ≤ 0.05 significance level.

## Results

### Intake and whole tract digestibility

There are no differences of intake between HE an LE animals for RFI (Table 3). The HE-RFI group showed marginally significant higher values for NDF and NFC digestibility (g/kg), (*P* = 0.06 and 0.08, respectively). The HE and LE-RFI did not differ for DM, OM, CP, ADF or EE digestibility. The BW mean was similar (~297 kg; *P* > 0.05) between the HE and LE-RFI groups during the study period.

**Table 3:** Intake^1^ and digestibility in dairy heifers classified as high (HE) and low efficiency (LE) for RFI, RG and FCE.

Traits (unit)	RFI^2^		SEM^7^	*P*-value^8^	RG^3^		SEM	*P*-value	FCE^4^		SEM	*P*-value
	HE^5^	LE^6^			HE	LE			HE	LE		
***Intake***												
C DM (kg/d)^9^	6.84	6.86	0.15	0.83	7.04	6.62	0.15	0.10	6.72	6.78	0.14	0.84
C DM (g /kg BW)	23.2	23.3	0.01	0.48	20.4	20.2	0.01	0.60	20.2	20.3	0.01	0.86
C DM (g /kg BW^0.75)^)	95.4	97.1	0.01	0.59	90.3	90.1	0.01	0.92	90.1	90.2	0.01	0.96
C OM (kg/d)^10^	6.40	6.40	0.11	0.99	6.48	6.25	0.11	0.25	6.25	6.29	0.12	0.12
C CP (kg/d)^11^	1.13	1.18	0.02	0.28	1.15	1.14	0.02	0.94	1.11	1.14	0.02	0.40
C ADF (kg/d)^12^	1.61	1.63	0.02	0.75	1.62	1.58	0.03	0.39	1.58	1.57	0.02	0.74
C NDF (kg/d)^13^	2.70	2.67	0.05	0.75	2.43	2.31	0.01	0.71	2.67	2.63	0.05	0.11
C EE (kg/d)^14^	0.17	0.18	0.01	0.60	0.17	0.18	0.05	0.23	0.16	0.18	0.01	0.11
***Digestibility (g/kg)***												
DM	747	734	5.42	0.21	747	740	6.00	0.59	745	728	6.48	0.20
OM	766	752	5.10	0.13	761	761	5.51	0.99	761	746	5.65	0.17
CP	785	783	5.54	0.90	783	789	6.25	0.63	780	776	6.16	0.76
ADF	608	587	11.3	0.37	592	596	12.03	0.87	597	571	10.9	0.25
NDF	634	603	8.85	0.06	623	621	9.45	0.95	622	596	9.56	0.57
EE	779	803	12.3	0.34	781	806	12.43	0.32	779	810	12.5	0.23
NFC^15^	906	899	2.18	0.08	905	903	1.92	0.66	906	894	2.50	0.01
BW (kg)^16^	300	294	4.18	0.50	305	279	4.57	<0.01	310	274	4.33	<0.01

^1^Dry matter intake data are restricted to the 5 d measurement period of the digestibility trial.

^2^Residual feed intake

^3^Residual weight gain

^4^Feed conversion efficiency

^5^High efficiency

^6^Low efficiency

^7^Stander error of the means

^8^Statistical difference between HE and LE

^9^Dry matter

^10^Organic matter

^11^Crude protein

^12^Acid detergent fiber

^13^Neutral detergent fiber

^14^Ether extract

^15^Non-fibrous carbohydrates

^16^Body weigh

The HE-RG animals showed a marginally significant higher DMI (*P* = 0.10) during the metabolism trial compared to LE-RG (7.04 *vs*. 6.62 kg/d). In the metabolism trial, HE-RG animals were 26 kg heavier compared to LE-RG animals (305 *vs*. 279 kg BW; *P* < 0.01; Table 3). The digestibility of DM and other components were similar between HE and LE groups for RG (*P* > 0.05).

The HE and LE-FCE showed no differences in DMI during the digestibility trial (*P* > 0.05; Table 3), similar to the evaluation of intake in the pre-experimental FE test (*P* > 0.05; Table 1). In the metabolism trial, HE-FCE animals were 36 kg heavier than LE-FCE animals (310 *vs*. 274 kg; *P* < 0.01). A higher NFC digestibility was obtained for HE-FCE in relation to the LE-FCE animals (906 *vs*. 894 g/kg, *P* < 0.01).

### Energy partitioning

**Fig 1:**
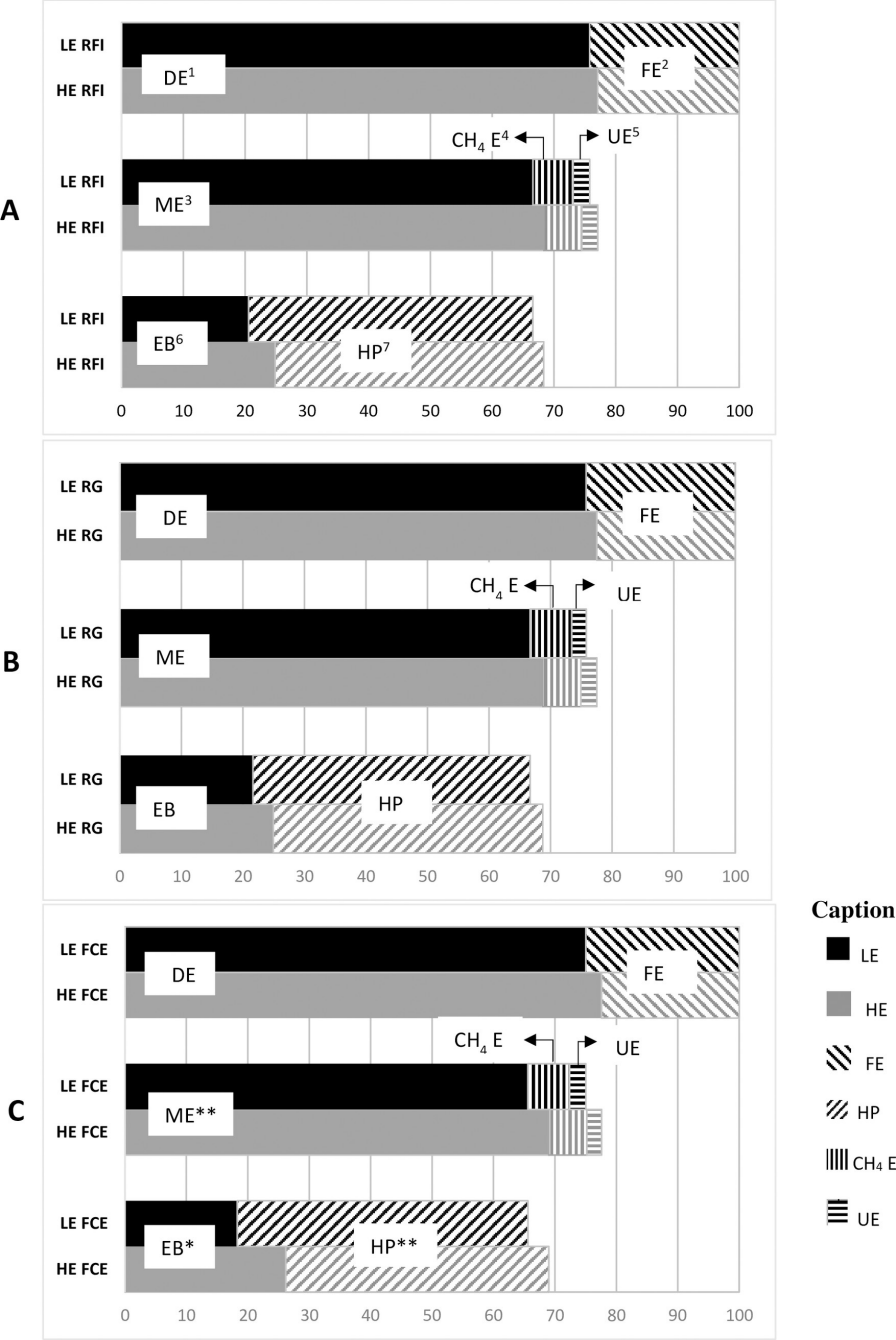
Energy partition in Holstein x Gyr heifers divergent for residual feed intake (RFI) figure A, residual weight gain (RG) figure B and feed conversion efficiency (FCE) figure C, indexes by high efficiency (HE) and low efficiency (LE), represented by black and gray bars, respectively. Indicative of significance in (*P ≤ 0.05 and **0.05 < P ≤ 0.10).

Since the DMI were similar between the HE and LE-RFI heifers during the metabolism trial, the GEI, DEI and MEI were also similar (*P* > 0.05), due to no differences in the energy losses in feces, urine and CH_4_, expressed as Mcal/d/BW^0.75^ (Table 4). Heat production was lower for the HE-RFI group (171 *vs*. 178 kcal/d/BW^0.75^; *P* = 0.05), however, the EB was similar. When expressed as percentages of GEI, there was no difference in energy partitioning between HE and LE-RFI groups (Fig 1). The ratio between digestible energy and gross energy (DE:GE), the metabolizability (ME:GE) and the ratio between metabolizable energy and digestible energy (ME:DE) did not differ between the HE and LE- RFI groups.

**Table 4:** Energy partitioning in dairy heifers classified as high (HE) and low efficiency (LE) for RFI, RG and FCE.

Traits	RFI^1^		SEM^6^	*P-*value^7^	RG^2^		SEM	*P-*value	FCE^3^		SEM	*P-*value
	HE^4^	LE^5^			HE	LE			HE	LE		
***Energy intake (Mcal/d/BW***^***0*.*75***^***)***												
Gross energy	0.40	0.39	0.01	0.50	0.41	0.39	0.01	0.47	0.41	0.38	0.72	0.11
Digestible energy	0.31	0.29	0.01	0.34	0.32	0.30	0.01	0.33	0.32	0.28	0.01	0.06
Metabolizable energy	0.27	0.26	0.01	0.31	0.28	0.26	0.01	0.30	0.28	0.25	0.01	0.06
***Energetic outputs***												
Feces (Mcal/d/BW^0.75^)	0.09	0.09	0.01	0.53	0.09	0.10	0.01	0.40	0.09	0.10	0.03	0.43
Urine (Mcal/d/BW^0.75^)	0.01	0.01	0.01	0.83	0.02	0.01	0.01	0.09	0.01	0.01	0.01	0.68
Methane (Mcal/d/BW^0.75^)	0.02	0.03	0.01	0.68	0.03	0.03	0.01	0.25	0.03	0.03	0.01	0.61
Heat production (kcal/d/BW^0.75^)	171	178	1.99	0.05	175	176	1.86	0.76	171	178	1.80	0.04
Energy balance (Mcal/d/BW^0.75^)	0.10	0.08	0.01	0.19	0.10	0.09	0.01	0.32	0.11	0.07	0.01	0.04
***Energy use efficiency***												
DE:GE^8^	0.77	0.76	0.01	0.31	0.78	0.76	0.01	0.28	0.78	0.75	0.01	0.15
ME:GE^9^	0.68	0.67	0.01	0.22	0.69	0.67	0.01	0.24	0.69	0.66	0.01	0.08
ME:DE^10^	0.85	0.84	0.01	0.24	0.85	0.84	0.01	0.56	0.85	0.84	0.01	0.09

^1^Residual feed intake

^2^Residual weight gain

^3^Feed conversion efficiency

^4^High efficiency

^5^Low efficiency

^6^Stander error of the means

^7^Statistical difference between HE and LE

^8^Ratio between digestible energy and gross energy

^9^Metabolizability

^10^Ratio between metabolizable energy and digestible energy

There were no differences in energy partitioning between HE and LE-RG groups (Table 4; Fig 1). The HE-RG showed a marginally significance to lose more energy in urine (0.02 *vs*. 0.01 Mcal/d/BW^0.75^; *P* = 0.09) than the LE-RG heifers.

The HE-FCE showed marginally significant (*P* = 0.06) higher DEI (0.32 *vs*. 0.28 Mcal/d/BW^0.75^) and MEI (0.28 *vs*. 0.25 Mcal/d/BW^0.75^) compared to LE-FCE animals. The LE-FCE group showed higher HP then HE-FCE animals (171 *vs*. 178 kcal/d/BW^0.75^, *P* = 0.04). The HE-FCE showed a difference in EB (0.11 *vs*. 0.07, *P* = 0.04), with higher energy retention for HE animals. The HE-FCE animals also showed a marginally significant higher ME:GE (0.69 *vs*. 0.66; *P* = 0.08) and ME:DE (0.85 *vs*. 0.84; *P* = 0.09) in relation to LE-FCE group. The percentages of GEI presented as metabolizable energy and HP were higher for the HE-FCE group (Fig 1) with marginally significance (*P* = 0.06). The most efficient animals for FCE showed greater retention of body energy (*P* = 0.04).

### Nitrogen balance

No differences were observed for nitrogen partition variables between HE and LE groups for RFI and RG (Table 5; *P* > 0.05). The comparison between HE and LE-FCE showed that the HE group excreted less urinary nitrogen (94.6 *vs*. 106 g/d; *P* = 0.02) compared to the LE group.

**Table 5:** Nitrogen partitioning in dairy heifers classified as high (HE) and low efficiency (LE) for RFI, RG and FCE.

Traits	RFI^1^		SEM^6^	*P*-value^7^	RG^2^		SEM	*P*-value	FCE^3^		SEM	*P*-value
	HE^4^	LE^5^			HE	LE			HE	LE		
Nitrogen Intake (g/d)	180	189	3.35	0.22	183	182	3.24	0.94	177	183	3.29	0.38
Feces nitrogen (g/d)	38.7	40.9	1.19	0.38	39.6	38.6	1.18	0.60	38.7	41.2	1.29	0.70
Digestible nitrogen (g/d)	142	148	2.85	0.29	143	144	2.92	0.94	138	142	2.86	0.55
Urinary nitrogen (g/d)	102	103	2.72	0.51	99.5	99.9	3.01	0.81	94.6	106	2.74	0.02
Retained nitrogen (g/d)	39.7	44.8	3.76	0.51	44.4	44.4	3.91	0.99	44.2	35.4	3.66	0.18
RN:Dig N^8^	0.27	0.30	0.02	0.48	0.30	0.30	0.02	0.96	0.30	0.24	0.02	0.20

^1^Residual feed intake

^2^Residual weight gain

^3^Feed conversion efficiency

^4^High efficiency

^5^Low efficiency

^6^Stander error of the means

^7^Statistical difference between HE and LE

^8^Ratio between retained nitrogen and digestible nitrogen

### Respiratory exchanges and methane emission

Animals from the HE-RFI (*P* = 0.04) and HE-FCE groups (*P* = 0.02) had lower O_2_ consumption (L/kg BW^0.75^) and lower CO_2_ production (L/kg BW^0.75^) in relation to their corresponding LE groups (Table 6). High efficiency animals for RFI presented a marginally significant lower CH_4_ intensity (g/kg BW; *P* = 0.08).

**Table 6:** Oxygen consumption and carbon dioxide and methane emissions in dairy heifers classified as high (HE) and low efficiency (LE) for RFI, RG and FCE.

Traits	RFI^1^		SEM^6^	*P*-value^7^	RG^2^		SEM	*P*-value	FCE^3^		SEM	*P*-value
	HE^4^	LE^5^			HE	LE			HE	LE		
***Respiratory exchanges***												
VO_2_ (L/kg BW^0.75^)^8^	33.9	35.3	0.30	0.04	34.6	35.0	0.28	0.72	34.0	35.5	0.28	0.02
VO_2_ (L/d)	2484	2525	32.9	0.46	2562	2425	31.9	0.02	2424	2547	29.3	0.12
VCO_2_ (L/kg BW^0.75^)^9^	35.6	37.5	0.38	0.05	36.3	36.9	0.36	0.58	35.4	37.7	0.37	0.02
VCO_2_ (L/d)	2607	2690	37.6	0.25	2689	2562	36.4	0.04	2523	2706	35.2	0.27
***Methane emissions***												
CH_4_ (g/d)^10^	130	136	2.48	0.19	134	127	2.50	0.27	129	134	2.46	0.20
CH_4_ (g/ kg BW)	0.4	0.5	0.01	0.08	0.4	0.5	0.01	0.47	0.4	0.5	0.01	0.73
CH_4_ (g/ kg BW^0.75^)	1.8	1.9	0.03	0.10	1.8	1.8	0.03	0.80	1.8	1.9	0.03	0.63
CH_4_:DMI (g/kg)^11^	19.3	19.8	0.49	0.76	19.5	20.8	0.48	0.33	19.7	20.7	0.52	0.53
CH_4_:OMI (g/kg)^12^	28.2	27.0	0.94	0.69	26.2	29.9	0.91	0.15	26.6	28.4	0.70	0.36
CH_4_:NDFI (g/kg)^13^	46.6	48.3	1.12	0.54	47.9	46.2	1.08	0.60	47.3	48.8	0.99	0.53
CH_4_:DDM (g/kg)^14^	20.6	21.4	0.48	0.53	21.2	20.5	0.48	0.63	21.1	21.3	0.46	0.83
CH_4_:DOM (g/kg)^15^	28.0	28.4	0.61	0.81	27.9	27.7	0.64	0.96	27.9	28.6	0.66	0.64
CH_4_:DNDF (g/kg)^16^	76.8	81.7	2.28	0.37	80.0	76.8	2.25	0.61	78.9	85.3	2.24	0.28
CH_4_:ADG (g/kg)^17^	153	157	2.78	0.63	142	169	3.45	0.01	147.1	163	3.07	0.04

^1^Residual feed intake

^2^Residual weight gain

^3^Feed conversion efficiency

^4^High efficiency

^5^Low efficiency

^6^Stander error of the means

^7^Statistical difference between HE and LE

^8^Volume oxygen

^9^Volume carbon dioxide

^10^Volume methane

^11^Ratio between methane and dry matter intake

^12^Ratio between methane and organic matter intake

^13^Ratio between methane and neutral detergent fiber intake

^14^Ratio between methane and digestible dry matter

^15^Ratio between methane and digestible organic matter

^16^Ratio between methane and digestible neutral detergent fiber

^17^Ratio between methane and average daily gain

For RG index, the HE animals consumed more O_2_ (*P* = 0.02) and produced more CO_2_ L/d, (*P* = 0.04) than LE group. The RG values showed a positive correlation (r = 0.32; *P* = 0.03) with the volume of O_2_ consumed (L/d). High efficient animals for RG and FCE showed 16% (*P* = 0.01) and 10% (*P* = 0.04) lower CH_4_ intensity (CH_4_:ADG) in relation to LE animals, respectively. The greater ADG for the most efficient animals for these FE indexes influenced the CH_4_ intensity (CH_4_:ADG).

### Blood metabolic variables

The divergent phenotype groups for RFI showed similar glucose, glucose:insulin ratio and non-esterified fatty acid (NEFA) concentrations (*P* > 0.05; Table 7). No differences were found for NEFA and glucose:insulin ratio between the divergent groups for RG (*P* > 0.05). Marginally significant higher glucose (*P* = 0.10) and insulin concentrations for HE-RG (*P* = 0.08) in comparison to LE-RG were observed. Glucose was higher in HE-FCE than LE-FCE (*P* = 0.01; 5.24 *vs*. 4.92 mmol/L). No differences were observed for insulin, glucose:insulin ratio, BHB and NEFA between HE and LE-FCE groups (*P* > 0.05).

**Table 7:** Hormonal and metabolites traits in dairy heifers classified as high (HE) and low efficiency (LE) for RFI, RG and FCE.

Traits (unit)	RFI^1^		SEM^6^	*P*-value^7^	RG^2^		SEM	*P*-value	FCE^3^		SEM	*P*-value
	HE^4^	LE^5^			HE	LE			HE	LE		
Insulin (pmol/L)	168	221	15.6	0.09	222	173	14.0	0.08	194	153	14.4	0.16
Glucose (mmol/L)	4.98	5.08	0.07	0.99	5.17	4.96	0.06	0.10	5.24	4.92	0.06	0.01
Gluc:Ins (mmol/L/pmol/L)^**8**^	0.03	0.03	0.01	0.92	0.03	0.03	0.02	0.53	0.03	0.04	0.04	0.30
BHB (mmol/L)^**9**^	0.80	0.88	0.02	0.03	0.82	0.80	0.02	0.78	0.81	0.79	0.02	0.66
NEFA (mmol/L)^**10**^	0.08	0.07	0.01	0.61	0.07	0.08	0.01	0.19	0.07	0.09	0.01	0.20

^1^Residual feed intake

^2^Residual weight gain

^3^Feed conversion efficiency

^4^High efficiency

^5^Low efficiency

^6^Stander error of the means

^7^Statistical difference between HE and LE

^8^Ratio between glucose and insulin ratio

^**9**^β-hydroxybutyrate

^**10**^Non-esterified fatty acids.

## Discussion

To test the hypothesis of the study “i) Despite of which FE index used to classify animals in HE and LE, the groups will present physiological differences that justify a better use of energy and protein in HE group, and ii) Classifications by different feed efficiency indexes will result in different physiological responses between animals classified HE and LE” we classified thirty-five heifers into HE or LE groups for feed efficiency indexes (RFI, RG and FCE). We performed a whole tract digestibility assay and gas exchanges study in open-circuit respiratory chambers to compare metabolic variables related to energy and protein use between FE divergent groups. The differences of intake, digestibility, energy and nitrogen partition, CH_4_ emission, blood metabolic variables and heat production between HE and LE groups varied according to FE index adopted. In general, animals classified as HE lost less N in urine (FCE) and energy as heat production (for RFI and FCE), showed less CH_4_ intensity (for RG and FCE) and presented higher positive energy balance (FCE), hence our hypothesis was proved.

### Intake and whole tract digestibility

The HE and LE-RFI showed no differences in DM, OM, CP, EE and ADF intake obtained during digestibility trial, corroborating our results, with observed in previous trials what did not find differences in digestibility between divergent groups for RFI [26,27]. The variation in the digestibility of feed explains around 10% the variation in RFI divergence [28]. There are some controversial results published about the effect of RFI classification on digestibility. Some studies have found that diet digestibility is negatively correlated with RFI [29,30] and others [31,32] showed no association between these two variables. The lack of effect in the present study suggests that ruminal digestibility have minor power to explain the divergence for RFI.

The HE-RG animals showed higher DMI with marginal significance during the metabolism trial compared to the LE-RG. A previous study, performed in Irland with 2,605 bulls showed that HE-RG animals had a slightly higher DMI than LE-RG animals [33].

In the present study, the HE-FCE and LE-FCE showed no differences in DMI during the digestibility trial, contrasting with other study[39], that showed higher DMI for HE-FCE compared with LE animals [34]. But these authors worked with middle lactation dairy cows and justified the higher DMI by higher fractional rate of passage associated with higher DMI. For the HE-FCE group in the present study was obtained a highest NFC digestibility, which may be desirable when most of the energy in these compounds is retained by ruminal microorganisms and used for growth.

The RG and FCE indexes are associated with faster growth and larger animals [14,35]. The highest ADG of the most efficient animals for RG and FCE explain BW differences between HE and LE animals, while other studies reported ADG tended to be greater for low-RFI heifers [36]. The independence of BW included in the multiple regression model for RFI and RG is one of the main advantages of these parameters. However, [37] reported that this independence does not necessarily imply genetic independence from mature cow BW because the genetic correlation between mature cow BW and BW from growing animals is not a unity. Overall, HE animals did not presented differences significant in digestibility between groups RFI and RG.

### Energy partitioning

The similarity of GEI, ME:DE, ME:GE and DE:GE between HE and LE groups for RFI, RG or FCE agree with the previous reports [28,38] that did not find differences in the proportion of energy lost as CH_4_ between divergent phenotypes for RFI. However, diverge from another study [29], where lower fecal and CH_4_ energy losses were observed.

There were positive correlations between FCE and DEI (r = 0.51; *P* < 0.01) and MEI (r = 0.52; *P* < 0.01) indicating that the efficiency of metabolizable and digestible energy use were greater for HE-FCE. Positive correlations were observed between FCE and EB (r = 0.52; *P* < 0.01) indicating that LE-FCE animals ate more and had greater energy losses (Table 8).

**Table 8:** Pearson correlations between productive performance in dairy heifers classified as high (HE) and low efficiency (LE) for RFI, RG and FCE.

Traits (unit)	RFI^1^		RG^2^		FCE^3^	
	r	*P-*value	r	*P-*value	r	*P-*value
***Digestibility***						
BW (kg)^**4**^	-	-	0.46	0.02	-	-
***Energy partitioning***						
Feces energy (Mcal/d/BW^0.75^)	-	-	-	-	-0.49	0.02
Digestible energy intake (Mcal/d/BW^0.75^)	-	-	-	-	0.51	0.01
Metabolizable energy intake (Mcal/d/BW^0.75^)	-	-	-	-	0.52	0.01
Energy balance (Mcal/d/ BW^0.75^)	-	-	-	-	0.52	0.01
***Nitrogen partitioning***						
Urinary nitrogen (g/d)	-	-	-	-	-0.56	0.01
***Respiratory exchanges***						
VO_2_ (L/kg BW^0.75^)^**5**^	0.29	0.05	-	-	-0.29	-0.29	0.05
-0.29
VO_2_ (L/d)	-	-	0.32	0.03	-	-
VCO_2_ (L/kg BW^0.75^)^**6**^	0.33	0.02	-	-	-0.42	0.01
VCO_2_ (L/d)	-	-	-	-	-0.31	-0.31	0.03
-0.31
***Methane emissions***						
VCH_4_ (g/kg BW^0.75^)^**7**^	0.32	0.03	-	-	-	-

^1^Residual feed intake

^2^Residual weight gain

^3^Feed conversion efficiency

^4^Body weigh

^**5**^Volume oxygen

^**6**^Volume carbon dioxide

^**7**^Volume methane

Energy loss as HP (kcal/d/BW^0.75^) was higher in LE-RFI and LE-FCE groups, by previous study [24] reported that HE-FCE animal lost 10% less energy as HP compared with LE-FCE cows. Supporting our results, findings from authors evaluating Angus, Hereford, Limousin, Gelbvieh and Charolais steers [29]; Angus and Charolais bulls [30], Brangus heifers [31], and Holstein x Gyr calves [39] also showed that HE-RFI animals had lower HP. One of the major factors that may explain the best FE is the lower energy expenditure associated with HP [29]. Higher HP can be associated to higher maintenance costs [31]. It is necessary to evaluate if the lower HP of HE animals are due the lower feeding caloric increment or to lower maintenance requirements. A lower caloric increment is expected for animals that have lower intake and similar performance [40].

The present study showed strong differences in energy partitioning between HE and LE-FCE heifers. These differences included lower HP and CH_4_ production and higher blood glucose concentration. Heat production differences in FCE could have been associated with differences in efficiency of chewing activity, rumen fermentation, or conversion of ME to net energy [34]. Heat production was estimated by indirect calorimetry from the gases exchanges of heifers fed *ad libitum*. Therefore, the value of HP is associated with maintenance functions, as well as energy lost during fermentation, digestion and processing of nutrients. We suggest that the index FCE can indicate difference promising to evaluate energy partitioning in dairy heifers, however, more studies should be made to confirm this hypothesis.

### Nitrogen balance

Better efficiency of nitrogen use for HE-RFI was expected, but there are no important differences between HE and LE-RFI. High efficiency animals for RG, showed marginally significance to lost less nitrogen in urine, compared to LE animals, probably due to the higher growth rate and to possible changes in the chemical composition (fat and protein) of the tissues. The HE-FCE heifers excreted less urinary nitrogen compared to the LE-FCE group, another study found similar urinary nitrogen excretion between divergent groups for FCE [34].

### Respiratory exchanges and methane emission

The HE animals for RFI and FCE had lower O_2_ consumption (L/kg BW^0.75^) and lower CO_2_ production expressed as L/kg BW^0.75^ in relation to the LE groups. High efficiency animals for RFI showed a marginally significance for lower CH_4_ as g/kg BW, probably due differences in generation of gases during ruminal fermentation, waste excretion and heat production. A previous trials conducted by our research team, animals of HE-RFI used a face mask method to evaluate gas exchanges in pre-weaning calves Holstein x Gyr was also had lower O_2_ consumption (L/d) and lower CO_2_ production expressed in L/d and did not observe show differences in CH_4_ production [41]. In the second study, assessing Gyr pre-weaning calves, using an open-circuit respiratory chamber there were no differences in gas exchange (VO_2_, VCO_2_, and VCH_4_) between HE and LE-RFI groups[39].

A positive correlation was found between RFI and O_2_ consumption (r = 0.29; *P* = 0.05), CO_2_ production (r = 0.33; *P* = 0.02) and CH_4_ (r = 0.32; *P* = 0.03) expressed as L/kg BW^0.75^ (Table 8). Indicating that most efficient heifers for RFI consume less O_2_ and consequently produce less CO_2_ and CH_4_. Previous studies with grazing dairy heifers divergent for RFI showed a correlation of 0.42 between DMI and CH_4_/d [42]. The same authors also observed that the CH_4_ emissions in g/d and g/kg BW^0.75^ did not differ between groups divergent for RFI, but the CH_4_ emission in g/kg DMI and CH_4_% GE was higher for LE-RFI heifers.

The HE-RG animals consumed more O_2_ per day and produced more CO_2_ per day (Table 6). The RG showed a correlation (r = 0.32; *P* = 0.03) with the volume of O_2_ consumed (L/d). The FCE had a correlation with O_2_ consumption per kg BW^0.75^ (r = -0.29; *P* = 0.05), CO_2_ production per kg BW^0.75^ (r = -0.42; *P* = 0.01) and per day (r = -0.31; *P* = 0.03). Previous studies found similar CH_4_ and CO_2_ emissions in high and low efficiency FCE lactating cows, but when expressed as CH_4_ or CO_2_ yield (CH_4_:DMI g/kg and CO_2_:DMI g/kg), the results were lower for HE-FCE cows [34]. In the present study, HE animals for RG and FCE showed 16% and 10% lower CH_4_ intensity (CH_4_:ADG) in relation to LE animals, respectively (Table 6). The greater ADG for the most efficient animals for these FE indexes (Table 1) influenced the CH_4_ intensity (CH_4_:ADG). Therefore, the use of more efficient animals for RFI, RG and FCE may be a strategy for reducing CH_4_ intensity in dairy heifers. Improving FCE could help reduce these emissions while maintaining current levels of production [43]. Methane production is associated with differences in DMI [8]. If variability in CH_4_ emissions is a component of differences in feed efficiency, selective breeding for more feed efficient animals could both reduce CH_4_ emissions and increase productivity.

### Blood metabolic variables

In the present study, the divergent phenotype groups for RFI showed similar glucose, and NEFA concentrations. Others authors also found no correlation between RFI with NEFA and glucose concentrations [17]. The lower BHB concentrations for HE-RFI animals, may be associated an underlying variation in energetic efficiency, other studies with heifers reported that BHB levels were a predictor of energy efficiency for FE indexes [36]. There are no difference for blood metabolic variables between LE and HE-RG and glucose concentrations was higher in HE-FCE. Blood BHB and glucose can be used as potential biomarkers for the identification of HE Holstein x Gyr (F1) heifers raised under tropical conditions for RFI and FCE, respectively.

### Considerations about the use of FE indexes

The use of FCE in dairy cows corresponds to the feed conversion ratio used in meat-producing animals; these are desirable for use in breeding programmes because they are easy to measure and conceptually uncomplicated. However, use of FCE as a selection criterion has limitations, for instance, selection for greater milk output increases the cow’s energy requirement, which cannot be met solely by increased feed intake, resulting in mobilization of body tissue to support the increased energy demand during lactation. Crossbreed heifers evaluated in our study were bred to produce milk in grazing systems of tropical climatic areas, as shown in S1 Table. The use of animals with high maintenance requirements is not desirable.

A common measurement of FE in dairy cattle is RFI, which differs from FCE as it is designed to estimate net feed efficiency or metabolic efficiency of the cow. The biological factors related to RFI showed that differences in intake behaviour often explains part of the differences [44]. There is considerable variation occurs in RFI in growing dairy heifers[8]. Understanding FE indexes during the growth phase can increase the progress in their use, assisting in animal selection decisions early in their productive life. This index needs to be more evaluated for crossbreed animals raised in tropical conditions, but since it is not related to the increase in adult weight, it could be a promising index for use in dairy cattle.

In principle, RG is similar to RFI except it regresses ADG in terms of feed intake and BW instead of regressing feed intake in terms of BW and ADG [33]. Hence, improved RG is, on average, associated with faster growth rates but is not associated with differences in feed intake. The improvement of growth in crossbreed cattle is desirable due to the limitation in precocity conferred by the zebu (*Bos taurus indicus*) contribution. The approach of summing RFI and RG each with equal weighting will improve the identification of well-performing animals with high FE; the weightings on the individual index traits need not necessarily be equal and could be modified by the end user [9].

The use of RFI, RG and FCE indexes resulted in groups with different metabolism and it should be taken into account in future research. However, which index has better potential to improve the selection of more efficient animals, will depend on the purpose of each selection programs.

## Final considerations

The differences in productive, nutritional, physiological and metabolic parameters among the HE and LE groups for RFI, RG and FCE varied according to the adopted FE index. Digestibility of DM, OM, CP, EE, NDF and ADF did not impacted differences in HE and LE groups for RFI, RG and FCE. This study indicates blood glucose and BHB parameters, may aid in the identification of HE heifers for RFI and FCE. The HE FCE group excreted less urinary nitrogen. There were no differences in energy partitioning for RG groups. Specifically, heat production (kcal/d/BW^0.75^) were lesser in the HE RFI and FCE groups. The reduction of CH_4_ intensity conferred by HE RG and FCE can be used as strategy in differences in FE in dairy heifers raised under tropical conditions. Future studies should explore whether the HP of divergent animals is related to feeding heat increment or to maintenance requirements.

## Supporting information

S1 Table (DOCX)
