# Supplementary material for: Grouping crossbred Holstein x Gyr heifers according to different feed efficiency indexes and its effects on energy and nitrogen partitioning, blood metabolic variables and gas exchanges
Source: PLoS One. 2020 Sep 11;15(9):e0238419. doi: 10.1371/journal.pone.0238419 (PMC7485853; doi:10.1371/journal.pone.0238419)
Supplement: S1 Table — (DOCX) [file pone.0238419.s001.docx]

**SUPPORTING INFORMATION**

**S1 Table. Individual classification of dairy heifers according to residual feed intake (RFI), residual weight gain (RG) and feed conversion efficiency (FCE) indexes by high efficiency (HE), intermediary and low efficiency (LE).**

| **Animal** | **RFI** | **RFI Group** | **RG** | **RG Group** | **FCE** | **FCE Group** |
| --- | --- | --- | --- | --- | --- | --- |
| 1 | 0.09 | LE-RFI | 0.16 | HE-RG | 0.19 | HE-FCE |
| 2 | -0.34 | HE-RFI | -0.03 | Intermediary | 0.18 | Intermediary |
| 3 | 0.36 | LE-RFI | -0.01 | Intermediary | 0.15 | LE-FCE |
| 4 | 0.31 | LE-RFI | -0.09 | LE-RG | 0.15 | LE-FCE |
| 5 | -0.06 | Intermediary | -0.04 | LE-RG | 0.16 | LE-FCE |
| 6 | -0.16 | HE-RFI | -0.03 | Intermediary | 0.16 | LE-FCE |
| 7 | -0.24 | HE-RFI | 0.07 | HE-RG | 0.18 | Intermediary |
| 8 | -0.29 | HE-RFI | 0.07 | HE-RG | 0.18 | Intermediary |
| 9 | 0.21 | LE-RFI | 0.05 | HE-RG | 0.17 | Intermediary |
| 10 | -0.21 | HE-RFI | -0.09 | LE-RG | 0.17 | Intermediary |
| 11 | -0.34 | HE-RFI | -0.16 | LE-RG | 0.15 | LE-FCE |
| 12 | 0.03 | Intermediary | 0.02 | Intermediary | 0.18 | Intermediary |
| 13 | 0.01 | Intermediary | -0.12 | LE-RG | 0.15 | LE-FCE |
| 14 | 0.07 | Intermediary | -0.09 | LE-RG | 0.15 | LE-FCE |
| 15 | 0.66 | LE-RFI | -0.04 | LE-RG | 0.14 | LE-FCE |
| 16 | -0.06 | Intermediary | 0.01 | Intermediary | 0.16 | LE-FCE |
| 17 | -0.08 | Intermediary | -0.01 | Intermediary | 0.18 | Intermediary |
| 18 | 0.34 | LE-RFI | -0.02 | Intermediary | 0.15 | LE-FCE |
| 19 | 0.23 | LE-RFI | -0.14 | LE-RG | 0.14 | LE-FCE |
| 20 | 0.02 | Intermediary | -0.04 | LE-RG | 0.20 | HE-FCE |
| 21 | -0.19 | HE-RFI | -0.09 | LE-RG | 0.18 | Intermediary |
| 22 | 0.26 | LE-RFI | 0.09 | HE-RG | 0.19 | HE-FCE |
| 23 | -0.12 | Intermediary | -0.06 | LE-RG | 0.17 | Intermediary |
| 24 | 0.37 | LE-RFI | 0.07 | HE-RG | 0.19 | HE-FCE |
| 25 | -0.15 | Intermediary | -0.02 | Intermediary | 0.20 | HE-FCE |
| 26 | 0.08 | LE-RFI | 0.01 | Intermediary | 0.18 | Intermediary |
| 27 | -0.26 | HE-RFI | 0.04 | HE-RG | 0.21 | HE-FCE |
| 28 | -0.25 | HE-RFI | -0.08 | LE-RG | 0.17 | Intermediary |
| 29 | 0.59 | LE-RFI | 0.01 | Intermediary | 0.16 | LE-FCE |
| 30 | -0.18 | HE-RFI | 0.05 | HE-RG | 0.20 | HE-FCE |
| 31 | -0.28 | HE-RFI | 0.16 | HE-RG | 0.22 | HE-FCE |
| 32 | 0.13 | LE-RFI | 0.08 | HE-RG | 0.21 | HE-FCE |
| 33 | -0.11 | Intermediary | -0.01 | Intermediary | 0.20 | HE-FCE |
| 34 | -0.02 | Intermediary | 0.21 | HE-RG | 0.25 | HE-FCE |
| 35 | -0.34 | HE-RFI | 0.03 | HE-RG | 0.21 | HE-FCE |

The original feed efficiency indexes were made based on SD, classified into groups: high efficiency (HE) and low efficiency (LE) for RFI, RG and FCE. The number of animals per treatment for HE group were (RFI ˂ 0.5 SD below the mean (n = 13) for RG and FCE > 0.5 SD above the mean (n = 11). For LE group were RFI > 0.5 SD above the mean (n = 10), for RG (n = 11) and FCE ˂ 0.5 SD below the mean (n = 12). The remaining animals were classified as intermediate and were not included in subsequent analyses.
